# Establishing a Markerless Genetic Exchange System for *Methanosarcina mazei* Strain Gö1 for Constructing Chromosomal Mutants of Small RNA Genes

**DOI:** 10.1155/2011/439608

**Published:** 2011-09-20

**Authors:** Claudia Ehlers, Dominik Jäger, Ruth A. Schmitz

**Affiliations:** Institut für Allgemeine Mikrobiologie, Christian-Albrechts-Universität zu Kiel, Am Botanischen Garten 1-9, 24118 Kiel, Germany

## Abstract

A markerless genetic exchange system was successfully established in *Methanosarcina mazei* strain Gö1 using the *hpt* gene coding for hypoxanthine phosphoribosyltransferase. First, a chromosomal deletion mutant of the *hpt* gene was generated conferring resistance to the purine analog 8-aza-2,6-diaminopurine (8-ADP). The nonreplicating allelic exchange vector (pRS345) carrying the *pac*-resistance cassette for direct selection of chromosomal integration, and the *hpt* gene for counterselection was introduced into this strain. By a pop-in and ultimately pop-out event of the plasmid from the chromosome, allelic exchange is enabled. Using this system, we successfully generated a *M. mazei* deletion mutant of the gene encoding the regulatory non-coding RNA sRNA_154_. Characterizing *M. mazei*Δ*sRNA*_154_ under nitrogen limiting conditions demonstrated differential expression of at least three cytoplasmic proteins and reduced growth strongly arguing for a prominent role of sRNA_154_ in regulation of nitrogen fixation by posttranscriptional regulation.

## 1. Introduction

*Methanosarcina mazei* strain Gö1 belongs to the methylotrophic methanogenic *Archaea* and, due to its role in methane production, is of high ecological relevance [1]. It serves as an archaeal model for investigating nitrogen stress responses, salt adaptation, methane production from different substrates, energy metabolism, as well as analyzing the role of small RNAs as regulatory elements in stress responses [2–7]. Although it only grows under strictly anaerobic conditions, the organism is genetically tractable and single colonies can be obtained on agar plates, a general requirement for genetic studies [8, 9]. However, genetic manipulation is restricted due to the fact that puromycin is the only selectable marker commercially available for methanoarchaea, which complicates generation of multiple mutations or even complementation experiments. Using *Methanosarcina acetivorans,* Metcalf and coworkers developed a so-called markerless exchange method using the *hpt* gene encoding hypoxanthine phosphoribosyltransferase as a counterselectable marker [10]. A Δ*hpt* strain which shows resistance towards the toxic purine analog 8-aza-2,6-diaminopurine (8-ADP) can be used for counterselection following integration of an nonreplicable plasmid containing the wild-type *hpt* gene and the desired mutation with flanking regions for recombination. The complete plasmid is integrated into the site of the desired mutation (pop-in) in the chromosome by a single homologous recombination event, making the strain sensitive to 8-ADP and allowing selection for puromycin resistance. The presence of 8-ADP permits selection for removal of the plasmid-based *hpt* gene (in concert with the vector backbone) by another single homologous recombination (pop-out) event. During this latter event, the gene of interest can be exchanged by the mutant construct [10]. Theoretically, allelic exchange takes place with a chance of 50% resulting in the desired mutant strain.

The goal of this study was to establish this method for *M. mazei *in order to allow markerless chromosomal deletion or point mutations of small regulatory RNA genes. To set up the system, a Δ*hpt* strain as well as the allelic exchange vector containing the wild-type *hpt* gene for counterselection was generated. To validate the method, we deleted the small noncoding RNA sRNA_154_. This sRNA has been identified in a genome wide RNA-seq screen and shown to be differentially transcribed dependent on nitrogen availability [7]. We suggest that sRNA_154_ plays a central role in nitrogen regulation in *M. mazei, *potentially adding another level of regulation to the known regulatory mechanism via the general nitrogen transcriptional repressor NrpR [11, 12]. sRNA_154_ is located in the intergenic region of MM3337 and MM3338 encoding a conserved and a hypothetical protein, respectively [7, 13]. A potential NrpRI operator (GGTA-N6-TACC) has been identified in the promoter region of sRNA_154_ gene implying that this small RNA is under direct control of the global nitrogen regulator NrpRI [7]. 

## 2. Materials and Methods

### 2.1. Bacterial Strains and Plasmids

Strains and plasmids used in this study are listed in Table 1. Plasmid DNA was transformed into *E. coli* according to the method of Inoue et al. [14] and into *M. mazei *using liposome-mediated transformation as described recently [8, 15].

### 2.2. Growth

*M. mazei* wild-type and mutant strains were grown in minimal medium under a nitrogen gas atmosphere in 5 or 50 mL closed growth tubes, which were incubated at 37°C without shaking [18, 19]. To screen on 8-ADP, however, the concentration of yeast extract in the minimal medium was reduced from 2 g/L to 0.5 g/L. In general, the medium was supplemented with 150 mM methanol or 25 mM trimethylamine (TMA) and 40 mM acetate as carbon sources and reduced with 2 mM cystein and 1 mM sodium sulfide. For nitrogen limited growth, ammonium was omitted from the media; molecular nitrogen in the gas phase served as sole nitrogen source [19]. In general, the *Methanosarcina* cultures were supplemented with 100 *μ*g/mL ampicillin to prevent bacterial contamination. For mutant selection, puromycin (5 *μ*g/mL) was added to the medium, for counterselection during markerless exchange the medium was supplemented with 8-ADP (20 *μ*g/mL). Growth was monitored by determining the optical density of the cultures at 600 nm (O.D._600_). *M. mazei* wild-type and mutant strains were grown on solid medium by carefully spreading the cells on 1.5% bottom agar containing 25 mM TMA as carbon source and incubated in an intrachamber incubator under a gas atmosphere consisting of 79.9% N_2_, 20% CO_2_, and 0.1% H_2_S. Mutants were selected by adding 5 *μ*g/mL puromycin or 20 *μ*g/mL 8-ADP (final concentration) to the agar. To identify positive pop-out mutants, single colonies derived in the presence of 8-ADP were streaked in parallel on plates complemented with puromycin and 8-ADP, respectively, to screen for puromycin sensitivity and 8-ADP resistance.

### 2.3. Construction of Plasmids

All primers used in this study are listed in supplementary Table  1. The plasmid for generating an *M. mazei htp* null mutant was constructed as follows: the sequences 800 bp down- and upstream of the *hpt* gene were amplified using chromosomal *M. mazei* DNA and the primer sets Mm 201 800 up.for/Mm 201 800 up/rev and Mm 201 800 down.for/Mm 201 800 down.rev, respectively. The PCR products obtained contained additional synthetic primer-mediated restriction sites which, for the 800 up stream product included a *Bam*HI at the 5′ end and *Eco*RI site at the 3′ end and for the 800 downstream fragment an *Eco*RI site at the 5′ end and *Kpn*I site at the 3′ end. Both fragments were restricted using *Bam*HI/*Eco*RI and *Eco*RI/*Kpn*I, respectively, and cloned into pBSK+ (Stratagene, La Jolla, Calif, USA) yielding plasmid pRS283. The allelic exchange vector for the markerless exchange was generated by amplifying the *hpt* gene from chromosomal *M. mazei* DNA using the primers Mm hpt for and Mm hpt rev with additional *Bam*H1 and *Xho*I sites, respectively. The PCR fragment was digested using *Bam*HI and *Xho*I and ligated to *Bam*HI and *Xho*I linearized pBSK+ to generate plasmid pRS311. In order to provide the *hpt* gene of pRS311 with a strong archaeal promoter, the known p*mcr* promoter of *Methanococcus voltae* [9] was cloned upstream of the gene. This was achieved by amplifying p*mcr* with the primers pmcr BamHI and pmcr XhoI using pRS207 [8] as template. The PCR product was cloned into TOPO-TA-cloning vector pDRIVE (Qiagen, Hilden, Germany) yielding plasmid pRS269. Digestion of pRS269 with *Bam*HI resulted in excision of the p*mcr* promoter that was cloned into the *Bam*HI site located directly upstream of the *hpt* gene of plasmid pRS311, resulting in pRS320. Finally, the 1.7 kbp *Eco*RI fragment from pRS204 containing the *pac*-cassette under the control of the constitutive promoter (p*mcr)* and terminator (t*mcr) *from the *mcr*-gene of *M. voltae* was cloned into the unique *Not*I site of pRS320, generating plasmid pRS345. This plasmid was used for markerless allelic exchanges by cloning the desired mutation into its unique *Apa*I site. To construct the *M. mazei *sRNA_154_ deletion mutant, approximately 1000 bp of the upstream-flanking region of the small RNA was amplified using the primer pair Mm s154_1 for and Mm s154_1 rev using genomic *M. mazei *DNA as template. The PCR product (937 bp) was digested with *Apa*I and *Xho*I (restriction sites provided by the primers; see underlined sequences) and ligated into the *Apa*I/*Xho*I-opened pMCL210 vector resulting in plasmid pRS606. A 1364 bp PCR product of the downstream region was generated by primers Mm s154_2 for and Mm s154_2 rev introducing an *Xho*I and a *Sma*I restriction site, respectively, which was subsequently cloned into an *Xho*I/*Sma*I-linearized pRS606, fusing the sRNA_154_ flanking region together and thereby deleting sRNA_154_. The plasmid was designated pRS631. The complete deletion construct was excised using *Apa*I and *Sma*I, treated with Mung Bean nuclease, and ligated into pRS345, which was linearized with *Apa*I followed by a Mung Bean nuclease treatment yielding plasmid pRS632. All constructs were verified by sequence analysis.

### 2.4. PCR Analysis for Mutant Strain Confirmation

Verification of sRNA_154_ deletion was performed using the primer pair Mm s154_1 and Mm s154_seq rev A ~250 bp product of the *bla* gene was amplified using the primers bla rev. and bla for. The primer pair pac1 and pac2 was used to generate a ~300 bp product of the *pac*-cassette. Generally, 2 ng of chromosomal DNA was used as template.

### 2.5. RNA Preparation and Northern Blot Analysis

Total RNA isolations and Northern blot analyses were performed essentially as described before [7], except that Isol-RNA Lysis Reagent (5 PRIME GmbH, Hamburg, Germany) was used for total RNA preparation.

### 2.6. Cell Extracts

*M. mazei* cell extracts were prepared as described previously [20]. 65 *μ*g of *M. mazei wild type, *Δ*hpt *and Δ*sRNA_154_* crude extracts were separated by 12.5% SDS-PAGE.

## 3. Results and Discussion

As mentioned above, markerless exchange of alleles originally developed for *M. acetivorans* was applied for *M. mazei* using the *hpt* gene as counterselection marker [10] and successfully generated a null mutant of the gene encoding sRNA_154_.

### 3.1. Settingup a Markerless Exchange System for *M. mazei*

**Figure 1:**
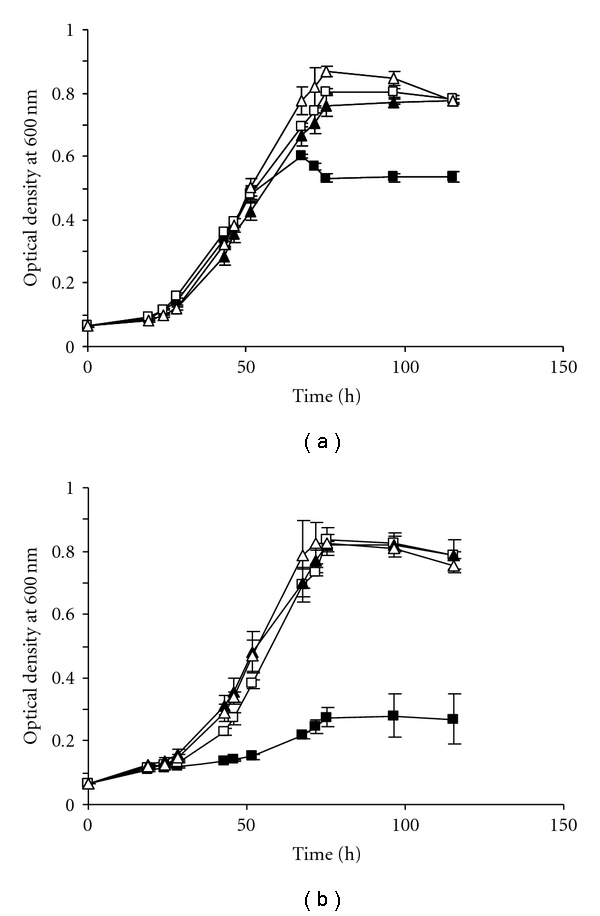
Growth analysis of *M. mazei *Δ*hpt* versus wild type in the presence of 8-ADP. Two different media were tested, (a) standard minimal medium [18] and (b) minimal medium with significantly reduced yeast extract (0.5 g/L). *M. mazei* strains were grown in 50 mL of the respective medium complemented with 150 mM methanol as carbon source under a gas atmosphere of N_2_/CO_2_ (80 : 20). Strains were incubated without additives (open symbols) or in the presence of 20 *μ*g/mL 8-ADP (closed symbols). Squares: wild type; triangles: *M. mazei *Δ*hpt. *Standard deviations of three replicates for each strain are indicated.

Members of the methanoarchaea become regularly resistant to the purine analog 8-ADP (2.9 × 10^−5^) [10], possibly by developing spontaneous mutations in the *hpt* gene that encodes a hypoxanthine phosphoribosyltransferase. Nevertheless, we decided to construct a Δ*hpt* mutant by applying the markerless exchange method of Pritchett et al. [10] rather than screening for a naturally occurring *hpt*-deficient strain. A pKS bluescript derivative was constructed carrying 800 bp of both the 5′ and 3′ flanking chromosomal region of the *M. mazei hpt* gene fused together, thereby creating an *hpt* deletion construct (pRS283). The nonreplicating plasmid pRS283 was transformed into M. mazei* [8], which will be referred to as wild type, and successful integration into the chromosome via a single homologous recombination event was confirmed by the gain of puromycin resistance (Figure S1A). Single colonies were then inoculated into liquid medium containing 20 *μ*g/mL 8-ADP. Cells that carry the wild-type *hpt* gene on the chromosome are sensitive to 8-ADP unless *hpt* is obliterated by a pop-out event, removing both the *hpt* gene and the plasmid backbone (Figure S1B). Unfortunately, the standard minimal medium used for *M. mazei* [18] cannot be used for this approach as 8-ADP had little effect on growth of the cells (Figure 1(a)).Yeast extract, which is presumably rich in purines and pyrimidines, might affect uptake of 8-ADP. Growth in media with significantly reduced yeast extract (0.5 g/L) clearly demonstrated that 20 *μ*g/mL 8-ADP was inhibitory (Figure 1(b)). As expected, the *M. mazei *Δ*hpt* grew in the presence of 8-ADP on standard and on yeast-reduced medium (Figures 1(a) and 1(b)). Single colonies of the *M. mazei *Δ*hpt* mutant strain were obtained by plating on solid medium containing 8-ADP, which were subsequently tested for puromycin sensitivity and simultaneous 8-ADP resistance by streaking on the respective plates. To confirm deletion of *hpt*, colonies that showed the desired phenotype were subjected to Southern blot analysis (data not shown). 

In a second step, the allelic exchange vector pR345 was constructed containing the *bla* gene and *pac*-resistance cassette for selection in *E. coli* and *M. mazei*, respectively, as well as the *hpt* gene as counterselectable marker. To provide the *hpt* gene with a strong promoter, the native promoter was exchanged with promoter p*mcr* of *M. voltae* [9]. The unique *Apa*I site in pRS345 provided an insertion site for the mutant construct of interest. 

### 3.2. Generation of a *M. mazei ΔsRNA_154_* Chromosomal Mutant

**Figure 2:**
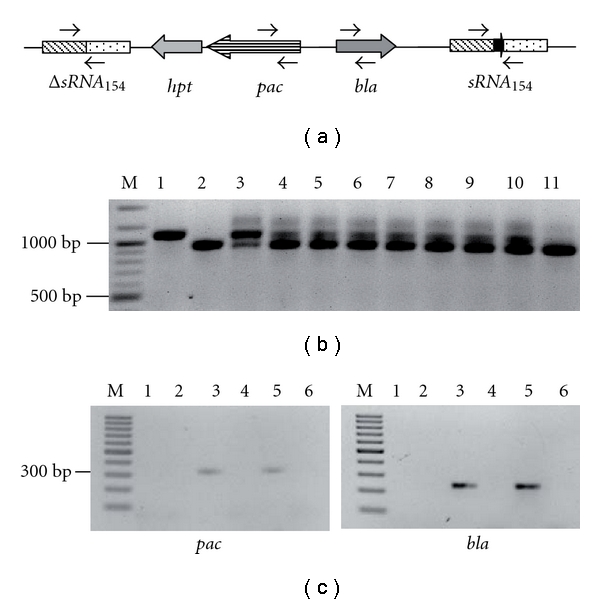
Verification of Δ*sRNA_154_* strains by PCR. (a) Cartoon of plasmid pRS623 integration into the *M. mazei* chromosome. Primers for PCR verification are shown by arrows (sizes do not correspond to actual proportions). (b) *M. mazei* wild-type and mutant strains Δ*sRNA_154_, *Δ*hpt,* and a diploid strain with pRS632 integrated into the chromosome were tested by PCR analysis for presence of the sRNA_154_ gene and ΔsRNA_154_ deletion construct. Lane M, 100 bp marker, Fermentas; lane 1: *M. mazei* wild type; lane 2: pRS623 carrying ΔsRNA_154_ deletion; lane 3: diploid strain; lanes 4–11: different Δ*sRNA_154_* candidate strains, whereas only the Δ*sRNA_154_* clone in lane 11 shows the unique genotype and was further analyzed by PCR analysis. (c) *M. mazei* wild-type and the same mutant strains were analyzed for presence of pRS632 backbone by PCR using primers for amplifying parts of the *bla* and *pac* genes, respectively. Lane M, 100 bp marker (Fermentas); lane 1: *M. mazei* WT; lane 2: Δ*hpt*; lane 3: diploid strain; lane 4: Δ*154*. lane 5: pRS632; lane 6: water control.

To validate the system, we deleted the gene encoding the small RNA_154_ which is transcribed exclusively under nitrogen limitation and supposedly plays a central role in nitrogen stress responses [7]. A deletion construct generated by fusing the flanking regions of sRNA_154_ together was inserted into the *Apa*I site of the allelic exchange vector pRS345. The resulting plasmid (pRS632) was transformed into the *M. mazei *Δ*hpt* strain followed by selection for pop-in/pop-out events as described above. Successful deletion of the sRNA_154_ gene was evaluated by PCR. PCR verification with primers binding up- and downstream of sRNA_154_ was performed yielding a PCR product of ~1,100 bp for wild type and ~940 bp for *M. mazei *Δ*sRNA_154_*. The PCR product representing the wild type was detected as expected in *M. mazei* wild type and the diploid strain with plasmid pRS632 inserted into the chromosome (Figure 2(a)). The respective amplicon for Δ*sRNA_154_* was clearly detected in the control (pRS632), in the diploid strain and was very prominent in all eight potential *M. mazei *Δ*sRNA_154_* mutants analyzed (Figure 2(b)). However, in seven out of the eight putative Δ*sRNA_154_* mutants, traces of PCR products corresponding to the product derived from sRNA_154_ wild type were also observed. This might be explained by the fact that several archaea have been demonstrated to possess multiple genome copies, as has been recently described by Soppa and coworkers [21]. They showed that *M. acetivorans* contains up to 17 copies dependent on the growth phase [21]. This polyploidy might result in incomplete allelic exchange with some of the chromosome copies remaining wild type. Since we could only confirm one out of eight mutant candidates, it appears that this difficulty occurs more often than anticipated when generating chromosomal mutants of *M. mazei*.

**Figure 3:**
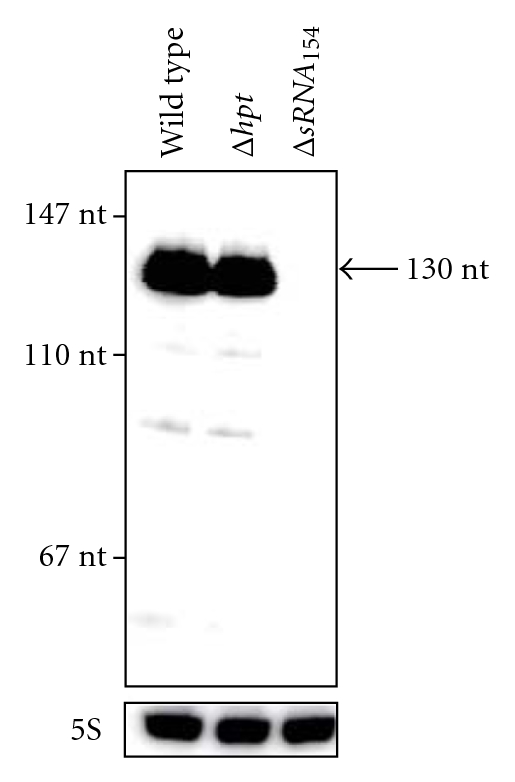
Northern blot analysis of *M. mazei *Δ*sRNA_154_* strain. Total RNA was purified from the respective *M. mazei *strains (Δ*sRNA_154_, *Δ*hpt, *and wild type) all grown under nitrogen limiting conditions. 10 *μ*g of each RNA were separated by a denaturing 6% PA gel and subsequently analyzed by Northern blot using a ^32^P-ATP-labelled oligonucleotide homologous to sRNA_154_. For each sample, the abundance of 5S rRNA was determined to exclude variations in RNA amounts.

The mutant depicted in lane 11 (Figure 2(b)) showing the Δ*sRNA_154_* PCR product was further examined for plasmid removal. PCR analyses using chromosomal DNA from *M. mazei* wild type, the Δ*hpt *mutant, the diploid strain, and Δ*sRNA_154_* as well as pRS632 as positive control clearly demonstrated the presence of the *bla* and *pac* genes exclusively in the diploid strain and the plasmid control, whereas for Δ*sRNA_154_,* both genes were not detectable. As a second line of evidence, Northern blot analyses were performed with total RNA derived from the wild type, Δ*hpt *and Δ*sRNA_154_* strains grown under nitrogen limitation and using a radioactively labelled oligonucleotide probe against sRNA_154_. Consistent with the previous data, Northern blot analyses clearly demonstrated that sRNA_154_ with a size of 130 nucleotides (nct) is present in the wild type and Δ*hpt* strains under nitrogen limitation but is not detectable in the *sRNA_154_* deletion strain, further confirming successful markerless allelic exchange (Figure 3). By generating this Δ*hptΔsRNA_154_* mutant, which will be referred to as Δ*sRNA_154_* strain, we have effectively established the markerless exchange system in *M. mazei*. 

### 3.3. Characterization of the *ΔsRNA_154_* Mutant Strain

**Figure 4:**
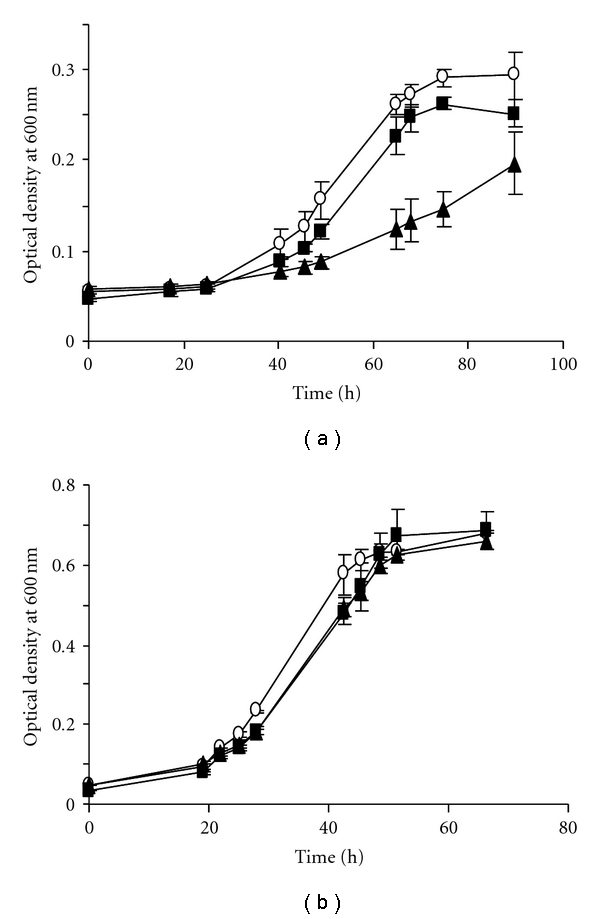
Growth analysis of Δ*sRNA_154_* versus wild type under nitrogen limitation (a) and under nitrogen sufficiency: (b) *M. mazei* strains were grown in 50 mL liquid minimal medium complemented with 150 mM methanol as carbon source under nitrogen limiting conditions (a) and with 10 mM NH_4_^+^ as nitrogen source (b) under a gas atmosphere of N_2_/CO_2_ (80 : 20). Open circles: wild type; closed squares: *M. mazei *Δ*hpt; *closed triangles:* M. mazei *Δ*sRNA_154_. *Standard deviations of five replicates for each strain are indicated.

To analyze the functional role of sRNA_154_ in nitrogen metabolism, we characterized the *M. mazei *Δ*sRNA_154_* mutant growing under conditions of nitrogen limitation, in which the sRNA is strongly expressed. Growth analyses demonstrated reduced growth of *M. mazei *Δ*sRNA_154_* with a growth rate of *μ* = 0.02 h^−1^ compared to *μ* = 0.03 h^−1^ obtained for the wild type (Figure 4(a)). Nevertheless, Δ*sRNA_154_* did not reach the same final cell densities as the wild type. Negative effects on nitrogen fixation due to the absence of the *hpt* gene were excluded by analysing growth behaviour of the parental strain (*M. mazei *Δ*hpt*) (Figure 4(a)). As expected, no different growth phenotype of these three *M. mazei* strains was observed under nitrogen sufficiency as under this condition the sRNA_154_ is not transcribed (Figure 4(b)).

**Figure 5:**
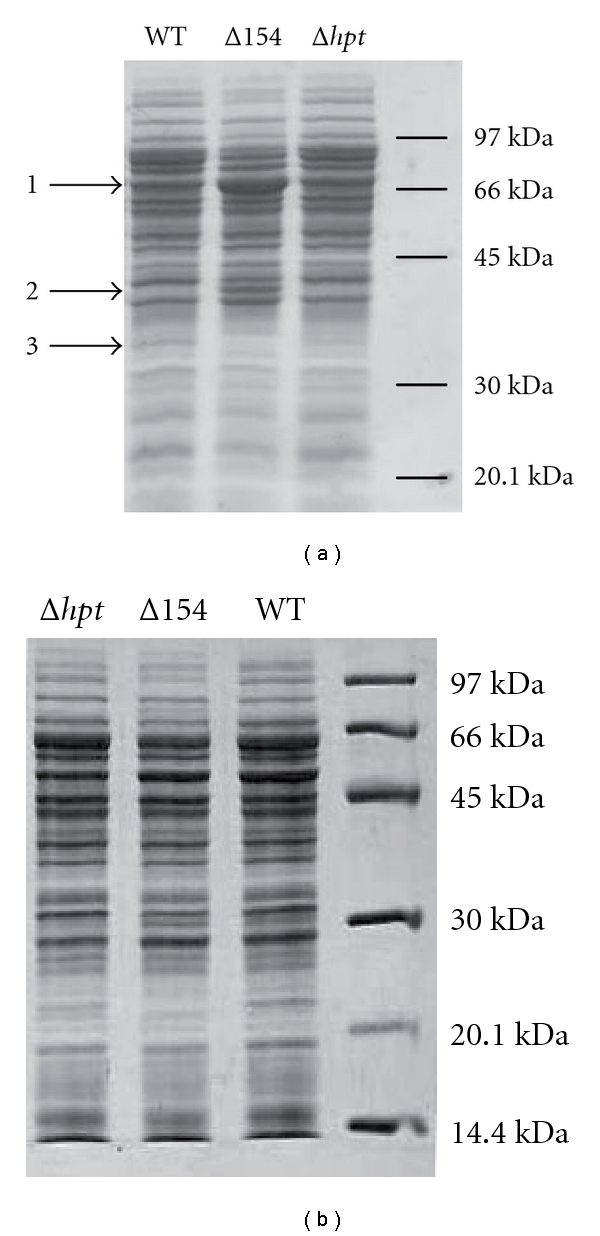
Analysis of protein expression pattern of Δ*sRNA_154_* versus wild type (a) under nitrogen limitation and (b) under nitrogen sufficiency. The *M. mazei* strains Δ*sRNA_154_, *Δ*hpt,* and wild type were grown in 50 mL medium under standard nitrogen-limiting conditions or under nitrogen sufficiency (see Figure 4). Cells were harvested in exponentially phase. Equal amounts of cell extracts (65 *μ*g) were applied to a 12% SDS-PA gel, which was subsequently stained with Coomassie blue. Arrows 1–3 indicate protein bands with differential expression in *M. mazei *Δ*sRNA_154_* and wild-type strains. Marker: LMW marker GE healthcare.

Characterizing the protein expression patterns of Δ*sRNA_154_* under nitrogen limitation and nitrogen sufficiency by one-dimensional SDS-PAGE clearly demonstrated differences in the protein patterns only under nitrogen depletion (Figure 5). At least three different proteins were differentially synthesized under nitrogen limitation in the absence of sRNA_154_ in comparison to the wild type (Figure 5(a)). Two proteins (1 and 2) with the molecular mass of approximately 66 and 40 kDa were exclusively or significantly more strongly expressed in the mutant, whereas a 35 kDa protein (3) was present in the wild type but appears to be absent in the mutant. These findings indicate that sRNA_154_ controls the protein expression either directly or indirectly and again strongly support a prominent function of the sRNA_154_ in nitrogen regulation. 

The Δ*sRNA_154_* mutant represents the first chromosomal deletion mutant of a small RNA in *M. mazei*. As it is only transcribed under nitrogen fixing conditions, presumably under the control of the global nitrogen regulator NrpRI [7], we suggest that sRNA_154_ plays a central role in regulation of nitrogen metabolism. The differences in the cytoplasmic protein patterns that result in reduced growth of Δ*sRNA_154_* under nitrogen fixing conditions argue for a prominent role of sRNA_154_ in regulation of nitrogen fixation. Posttranscriptional regulation by sRNA_154_ would add another level of regulation of nitrogen metabolism in *M. mazei* possibly resulting in tighter control or fine tuning of translation of the target mRNAs. 

## 4. Conclusion

By generating a Δ*hpt* strain and a plasmid for allelic replacements, we successfully applied the markerless exchange system to *M. mazei*. The method was further optimized by using medium with reduced yeast extract, thereby enhancing the toxic effect of 8-ADP during counterselection. Generation of Δ*sRNA_154_* revealed the role of sRNA_154_ in nitrogen metabolism as demonstrated by reduced growth as well as differential synthesis of at least three proteins under nitrogen fixing conditions in the absence of sRNA_154_. 

## Figures and Tables

**Table 1:** Strains and plasmids used in this study.

Strain or plasmid	Genotype or description	Source or reference
Strains		

*Methanosarcina mazei* strain Gö1	Wild type	DSM No. 3647
*M. mazei *Δ*hpt*	*M. mazei*, with *hpt* deletion	This study
*M. mazei *Δ*sRNA_154_*	*M. mazei*, with sRNA_154_ deletion	This study
*E. coli* DH5*α*	general cloning strain	Stratagene, La Jolla, US
*E. coli* DH5*α/λ*pir	general cloning strain	[16]

Plasmids		

pMCl210	general cloning vector	[17]
pBSK+	general cloning vector	Stratagene, La Jolla, US
pDRIVE	general cloning vector	Qiagen, Hilden, Germany
pRS207	*pac*-resistance cassette in pSL1180	[8]
pRS269	p*mcr* of *M. voltae* in pDRIVE	This study
pRS283	*M. mazei hpt *deletion construct in pBSK+	This study
pRS311	pBSK+ plus *M. mazei hpt* gene	This study
pRS320	pRS311 with p*mcr* upstream of *hpt *	This study
pRS345	pRS311 with *pac*-resistance cassette	This study
pRS606	pMCL210 with 930 bp of sRNA_154_ upstream region	This study
pRS631	pMCl210 plus sRNA_154_ deletion construct	This study
pRS632	Δ154 construct (pRS631) inserted into the *Apa*I site of pRS345	This study
